# Identification and analysis of tumour-associated antigens in hepatocellular carcinoma

**DOI:** 10.1038/sj.bjc.6602460

**Published:** 2005-03-08

**Authors:** Y-Y Shi, H-C Wang, Y-H Yin, W-S Sun, Y Li, C-Q Zhang, Y Wang, S Wang, W-F Chen

**Affiliations:** 1 Immunology Department, Peking University Health Science Center, Beijing 100083, China; 2 Immunology Department, School of Medicine, Shandong University, Jinan, China; 3 Sun Yat-Sen University Cancer Center, Guangzhou, China; 4 Cancer Biological Therapy and Diagnosis Center, Beijing Cancer Hospital, Beijing, China; 5 Department II of Surgery and Laboratory of Surgical Oncology, Peking University People's Hospital, Beijing, China

**Keywords:** hepatocellular carcinoma, serological analysis of recombinant cDNA expression libraries, tumour antigens, cDNA microarray

## Abstract

To identify tumour and tumour-associated antigens in patients with hepatocellular carcinoma (HCC) one may find potential diagnostic markers and immunotherapeutic targets. In the current study, 30 distinct antigens reactive with serum IgG from HCC patients were identified by serological analysis of cDNA expression libraries (SEREX). The mRNA expression patterns of 14 of these 30 antigens were altered in cancer as further revealed by cDNA microarray, with upregulation for nine and downregulation for five antigens. One of the upregulated antigens was cancer-testis (CT) antigen (CAGE), which had been previously reported to be expressed exclusively in normal gametogenic tissues and aberrantly expressed in a variety of cancer cells. In our study, CAGE mRNA was expressed in 39.4% of HCC patients, 73.3% of patients with gastric cancer and 30.8% of patients with colorectal cancer. Antibodies against CAGE protein were detected in approximately 5.1% of the sera from HCC patients, 8.3% of that from gastric cancer patients and 7.3% of that from colorectal cancer patients. The relative high incidence of CAGE in cancer cells makes it a potential target for vaccine design. Another antigen of great interest is transgelin 2. The overexpression of transgelin 2 mRNA in a large per cent (69%) of HCC points to its potential as a diagnostic marker for HCC.

The mortality rate of hepatocellular carcinoma (HCC) is extremely high in China and ranks the third in malignant tumours worldwide with a global incidence of 1.2 million new cases per year. Surgery remains the most effective treatment for HCC. However, only 20% of HCC patients can be resected and the recurrence in resected patients is as high as 70%. Overall, the 5-year survival for resected patients is no more than 19%. As for therapies using chemical or physical approaches, the outcome is still poor because of the resistance of cancer cells in spite of some recent advances. (Butterfield and Ribas, 2002).

Antigen-specific immunotherapy is an alternative approach for the treatment of HCC (van der Bruggen *et al*, 1991). Over the past decade, a number of clinical trials have been conducted to examine the potential for the activation of tumour-specific T cells in HCC patients, many have shown biological activity and a subset has shown antitumour efficacy (Iwashita *et al*, 2003; Butterfield, 2004; Kuang *et al*, 2004). Recent reports on clinical trial of AFP-based vaccine indicated that spontaneous AFP-specific immunologic responses had been induced in vaccinated HCC patients. Further study on AFP-based immunotherapy is ongoing (Butterfield *et al*, 2003; Butterfield, 2004). Screening for potent tumour antigens capable of inducing vigorous T-cell response and blocking of tumour escape are the two major tools to improve the effectiveness of tumour antigen vaccines. Rapid identification of tumour-associated antigens by cytotoxic T-cell and antibody-based screening of cDNA expression libraries has provided attractive targets for cancer-specific immune response, which accelerates the progress of antigen-specific immunotherapy.

Cancer-testis (CT) antigens, which express in a wide range of different cancer types but not in normal tissues except for testis, ovary and placenta, are considered as tumour-specific shared antigens (Scanlan *et al*, 2002a). The category of CT antigens is the common target for tumour immunotherapy in clinical trials, for example, NY-ESO-1 is a potent CT antigen and its eliciting integrated cellular and humoral responses have been observed in vaccinated patients in clinical trials, with little or no toxicity (Jager *et al*, 2000; Chen *et al*, 2004; Davis *et al*, 2004). The spontaneous CD8^+^ T-cell response to HLA-A2-restricted NY-ESO-1b peptide in HCC has been reported by our group (Shang *et al*, 2004), and our application for clinical trial of NY-ESO-1b peptide vaccine for the immunotherapy in HCC patients is under way.

In an attempt to screen for potent tumour antigens expressed in HCC, serological analysis of cDNA expression libraries (SEREX) has been used to screen a newly constructed HCC expression library. A subset of 30 antigens was found to be associated with an HCC-related serological response, and 14 of these 30 antigens showed altered mRNA expression in HCC. The identification of these tumour antigens provided candidates for immunological applications and the molecular features for understanding tumorigenesis.

## MATERIALS AND METHODS

### Tissue specimens and sera

The cancer tissues, adjacent noncancerous tissues and sera were collected from Peking University teaching hospitals and the Resource Bank of Cancer Center, Sun Yat-Sen University with the informed consent of patients. Tissue samples were resected and frozen immediately in liquid nitrogen. Serum samples were collected and reserved at −70°C.

### Construction and immunoscreening of cDNA expression library

Total RNAs were extracted from two moderately differentiated HCC specimens with Trizol reagent (Life Technologies, Gaithersburg, MD, USA), and then mRNAs were purified by the poly-(A) Track kit (Promega, Madison, WI, USA) and mixed at an equal ratio. The HCC library was constructed and screened as described before (Wang *et al*, 2004).

### DNA sequencing

The cDNA inserts were sequenced using the BigDye Terminator Cycle Sequencing kit (PE Applied Biosystems, Foster City, CA, USA). The cDNA sequences were analysed using Genbank database (http://www.ncbi.nlm.nih.gov).

### cDNA microarrays

Two independent pools of RNA from tumours and the corresponding noncancerous tissues were prepared by mixing aliquots of RNA isolated from cancer tissues of six well-differentiated HCC and their adjacent noncancerous tissues, respectively. Another two similar pools were prepared using RNA isolated from cancer tissues of six poorly-differentiated HCC and their adjacent noncancerous tissues, respectively. Each pool was composed of an equal amount of all six samples. The microarray hybridisation was performed at the Gene Company Limited using the Human Genome U133A GeneChip array and the Affymetrix GeneChip platform. Analysis of microarray data was performed with the software package of Affymetrix. In this study, the differential expression was considered as significant between HCC and adjacent noncancerous tissues when the ratio of signals between the same spots on different membranes was greater than 2, with 95% confidence (*P*⩽0.5).

### Real-time quantitative reverse transcript-polymerase chain reaction analysis (RT–PCR)

The quantitation of transcripts from 16 paired HCC and their adjacent noncancerous tissues was performed by real-time quantitative reverse transcript-polymerase chain reaction (RT–PCR) (Schmittgen *et al*, 2000). Primers were designed using PrimerBank Database (Wang and Seed, 2003). The emission intensity of SYBR Green I bound to double-strand DNA was detected with the icycler system (Bio-Rad, Hercules, CA, USA). After an initial denaturation at 95°C for 3 min, the PCR reactions were cycled 40 times as follows: 5 s at 95°C, 10 s at the appropriate annealing temperature and the extension duration at 72°C is based on the length of the target sequence. Fluorescence intensity was measured at the end of each elongation phase. The melting curve analysis was carried out immediately after amplification, following the manufacturer's instructions. The fold change in target gene relative to G3PDH endogenous control was expressed as 2^−ΔΔCt^, where ΔΔCt=(Ct_Target_−Ct_G3PDH_)_T_−(Ct_Target_−Ct_G3PDH_)_N_. T represents cancerous tissue and N represents adjacent tissue.

### RT–PCR analysis

The expression frequency of CAGE gene was determined by RT–PCR analysis. Total RNA from cancer tissues and paired adjacent noncancerous tissues was extracted and treated with RNase-free DNase before reverse transcription with Advantage RT for PCR kit (Clontech, Palo Alto, CA, USA). RT–PCR of CAGE was performed with 35 cycles of 30 s at 94°C, 25 s at 60°C and 30 s at 72°C, followed by 7 min at 72°C. The sequences of paired primers were as follows: CAGE sense, 5′-ccaaagcaacaagctgcatgtc-3′; CAGE antisense, 5′-tcattcagcctcccaggagttg-3′.

### Western blot analysis

The serological reactivity of CAGE antigen was analysed by Western blot assay. The cDNA of CAGE was constructed into pQE31 expression vector, which was then used to transform *Escherichia coli* strain M15. The recombinant CAGE protein was induced by 1 mM IPTG. Purification of the CAGE protein with 6 × His tag was performed using Ni-NTA affinity chromatography (Qiagen).

The purified CAGE protein was resolved on 12% SDS–PAGE, and then transferred onto a nitrocellulose membrane. The nitrocellulose membrane was incubated with sera (1 : 500 dilution) for 1 h after blocking with 5% low-fat milk. After washing, the NC membrane was incubated with alkaline phosphatase-conjugated goat anti-human IgG (1 : 15000 dilution) for 1 h, and then processed for NBT/BCIP colour development.

## RESULTS

### SEREX defined HCC-associated antigens

Using SEREX to screen a newly constructed HCC cDNA expression library, 52 positive clones were obtained. Sequencing analysis showed that the 52 clones represented 30 distinct antigens (Table 1). Of these 30 antigens, one is the CT antigen CAGE protein, and the other 13 antigens, which had previously been reported to be involved in tumorigenesis or suspected to be involved in tumour progression, including CDC37 (Stepanova *et al*, 2000), MIF (Li *et al*, 2004), galectin 4 (Huflejt and Leffler, 2004), galectin 8 (Bidon-Wagner and Le Pennec, 2004), PINCH (Wang-Rodriguez *et al*, 2002), SPRY2 (Lee *et al*, 2004a), HSPCA (Becker *et al*, 2004; Huang *et al*, 2004b), transgelin 2 (Shields *et al*, 2002), HDAC2 (Zhu *et al*, 2004), H factor (Junnikkala *et al*, 2000), AAT (Huang *et al*, 2004a), B factor (Perou *et al*, 1999), PIBF (Lachmann *et al*, 2004). Three other antigens (SR140 protein, SFRS2IP, RNPC2) may be involved in the regulation of alternative mRNA splicing, PSMA7 is related to hepatitis B and hepatitis C viral replication (Zhang *et al*, 2000; Kruger *et al*, 2001), and the remaining 12 antigens have no known association with cancer or hepatitis B or hepatitis C.

### Expression patterns of the mRNA transcripts encoding tumour antigens identified by SEREX from HCC

**Figure 1:**
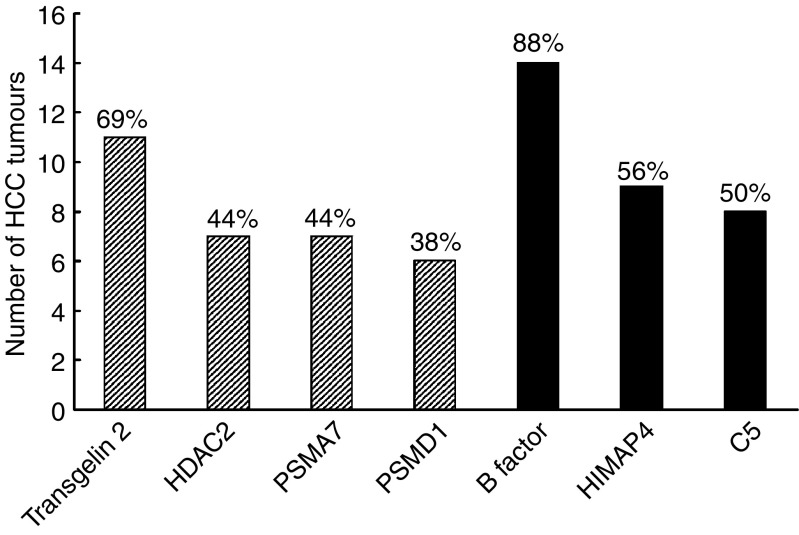
Seven differentially expressed antigens in HCC tissues measured by real-time quantitative RT–PCR. The frequencies for each altered gene out of 16 paired HCC are indicated on the top of each graphic bar (shadowed bars: overexpressed genes; solid black bars: downregulated genes).

With the intention to identify HCC tumour markers, expression patterns of the mRNA transcripts encoding serologically defined HCC antigens in HCC samples were evaluated by cDNA microarray analysis. Of the 30 antigens, 14 antigens showed differential mRNA expression in HCC samples relative to the adjacent noncancerous tissues. The mRNA expression levels of nine antigens, CAGE, transgelin2, HDAC2, RNPC2, PSMD1, HSPCA, PSMA7, U2-associated SR140 protein and galectin 4, were upregulated in HCC, whereas those of the other five antigens, HIMAP4, H factor, C5, AAT and B factor, were downregulated. To validate the cDNA microarray results, with the exception of CAGE, as well as HSPCA and galectin 4, which have been already reported to be overexpressed in HCC (Huang *et al*, 2004b; Huflejt and Leffler, 2004), the mRNA expression levels of seven antigens out of the remaining 11 antigens were further measured by real-time quantitative RT–PCR. Altered mRNA expression was defined as two-fold differences in the expression level in HCC relative to paired adjacent noncancerous tissue. Figure 1 summarised the quantitative RT–PCR results from 16 pairs of HCC tissues. Three antigens including B factor, HIMAP4 and C5 had downregulated mRNA expression in HCC relative to the adjacent noncancerous tissue, at frequencies of 88, 56 and 50%, respectively, and four other antigens including transgelin 2, PSMA7, HDAC2 and PSMD1 were overexpressed at frequencies of 69, 44, 44 and 38%, respectively. Of note, transgelin 2 was overexpressed at a high rate (69%) in HCC specimens.

### Expression of CAGE in different tumour entities

**Figure 2:**
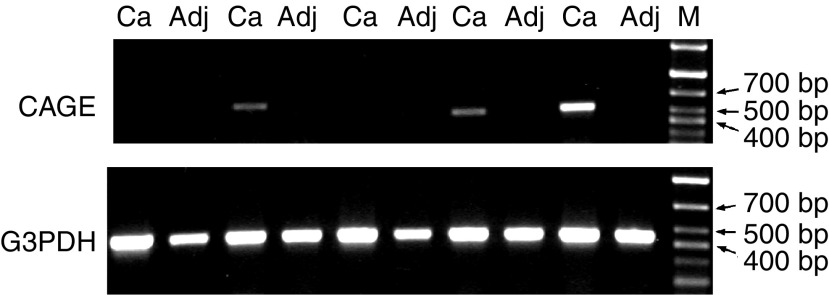
Expression of CAGE in representative HCC samples and paired adjacent noncancerous tissues by RT–PCR. CAGE was detected in three HCC tissues as a 529-bp PCR product. The expression of CAGE was negative in five paired adjacent noncancerous tissues. RT–PCR for G3PDH was used to monitor the quality of the RNA sample with a 452-bp PCR product (Ca: cancerous tissues; Adj: adjacent noncancerous tissues; M: marker).

CAGE is a CT antigen identified from a testis library by Cho *et al* (2002). To investigate the distribution of CAGE mRNA in HCC, the cancer tissues and paired adjacent tissues were examined by RT–PCR. CAGE mRNA was expressed in 39.4% (13 of 33) of HCC tissues and in 9.1% (three of 33) of HCC adjacent noncancerous tissues. Representative results are shown in Figure 2. In gastric cancer, CAGE mRNA was detected in 73.3% (11 of 15) of cancerous tissues and in 33.3% (five of 15) of noncancerous tissues. In colorectal cancer, the CAGE mRNA expression rate was 30.8% (four of 13) and 15.4% (two of 13), respectively.

### Serological reactivity of recombinant CAGE protein in cancer patients

**Figure 3:**
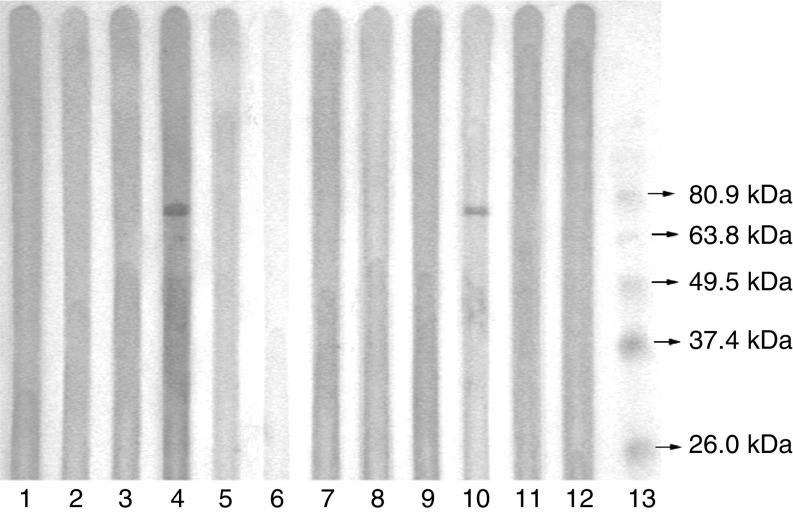
Serological reactivity of the recombinant CAGE protein in HCC patients by Western blot assay. Lane 13: protein marker; lanes 11–12: negative controls with normal sera; lane 10: positive control with the pooled sera, with which the HCC cDNA expression library was immunoscreened; lane 4: positive reaction with an HCC serum; lanes 1–3 and 5–9: negative reaction with HCC sera.

To determine the anti-CAGE antibodies produced in tumour patients, sera collected from patients with HCC, gastric cancer or colorectal cancer were screened by Western blot assay with the recombinant CAGE protein. Positive reaction was detected in 5.1% (four of 79) of HCC patients. Clinical histopathological diagnoses were available for three of the four patients who had serum antibody. Two patients were at stage II and one patient at stage III. The percentage of positive reaction was 8.3% (three of 36) in gastric cancer and 7.3% (three of 41) in colorectal cancer, and all these six patients had the cancer at stage III. Representative results were shown in Figure 3.

## DISCUSSION

SEREX has been proved to be an easy and effective approach for the identification of tumour antigens, and so far about 1200 SEREX-defined tumour antigens have been identified. Given that the size of the SEREX-defined cancer immunome, the repertoire of tumour antigens capable of eliciting immune response in cancer patients, is estimated to have 4000 antigens, continued efforts in SEREX screening should extend our understanding of tumour antigens (Lee *et al*, 2004b). With the aim to identify potent tumour-associated antigens for clinical applications, the SEREX screening of HCC cDNA expression library was performed in several laboratories (Stenner-Liewen *et al*, 2000; Wang *et al*, 2002; Uemura *et al*, 2003). These findings imply that different tumour-associated antigens may be identified by SEREX from distinct HCC samples. In this study, we established a pooled cDNA library from two patients with HCC and screened it with pooled sera. A total of 30 antigens reactive with serum IgG from HCC patients were identified, which include 14 antigens reported to be involved in tumorigenesis or suspected to be related to tumour progression. Of these, one is CT antigen (CAGE), which was first reported to be expressed in gastric cancer by Cho *et al* (2002) and found to be hypomethylated in the majority specimens of human HCC (Cho *et al*, 2003). Intriguingly, none of the 30 antigens overlapped with the antigens detected by the other two groups (Stenner-Liewen *et al*, 2000; Uemura *et al*, 2003), and only one of them (HCA58) was identified in our previous report (Wang *et al*, 2002).

Knowledge in the immunogenicity and expression pattern of serologically defined tumour antigens is critical in assessing their relevance to cancer and their therapeutic and diagnostic potentials. CT antigens, with restricted expression in normal gametogenic tissues and aberrantly expressed in a wide range of cancers, are considered to be ideal targets for cancer vaccination today. In the present study, the expression of CT antigen CAGE in HCC was first identified by antibody-based screening of SEREX and subsequently found to be overexpressed at mRNA level in 39.4% (13 of 33) of HCC specimens, 73.3% (11 of 15) of gastric cancer samples, and 30.8% (four of 13) of colorectal cancer samples. Antibody response was detected in 5.1% (four of 79) of the HCC patients, 8.3% (three of 36) of the gastric cancer patients and 7.3% (three of 41) of the patients with colorectal cancer. Thus, we have confirmed the high expression of CAGE mRNA in gastric cancer as reported by Cho *et al* (2002, 2003), and revealed the intermediate expression of CAGE in HCC and colorectal cancer. We also demonstrated that CAGE was immunogenic to elicit antibody response in cancer patients. As a CT antigen, CAGE might be a potential candidate for tumour vaccine design, provided it is proved that CAGE can induce T-cell responses.

We are aware that CAGE was detected to be expressed in minor but substantial number of adjacent noncancerous tissue specimens, 9.1% (three of 33), 33.3% (five of 15) and 15.4% (two of 13) in adjacent noncancerous tissues of HCC, gastric cancer and colorectal cancers, respectively. To define the cell types expressing CAGE mRNA in the adjacent noncancerous tissues, *in situ* hybridisation and histochemical staining were performed in the consecutive slides of the HCC adjacent noncancerous tissues with CAGE mRNA expression. Our results showed that these noncancerous tissues were in cirrhotic condition and some of the cirrhotic hepatocytes expressed CAGE mRNA (data not shown). As the cirrhosis was considered to be precancerous condition (Kanai *et al*, 2004), the positive signal of CAGE mRNA in cirrhotic hepatocytes might imply that these cells are at the transitional stage towards the HCC. To ensure whether CAGE was expressed in cirrhosis tissues, 19 cirrhosis samples were collected for further analysis of the expression of CAGE mRNA, and two of the 19 samples were observed to be positive (data not shown). A recent study by Cho and collegues has provided evidence for promoter hypomethylation of CAGE in the premalignant stage of gastric carcinoma and HCC (Cho *et al*, 2003). It is perceivable that CAGE is associated with the progression of tumorigenesis. Thereby, it may be worthwhile monitoring CAGE mRNA expression in patients with liver cirrhosis and observing the probability to develop into HCC.

In conformity with previous reports (Scanlan *et al*, 2002b; Wang *et al*, 2002) that a subset of antigens with cancer-related serological profile was encoded by the genes of altered expression, in this study, 14 of the antigens associated with an HCC-related serological response showed altered levels of mRNA expression, including five antigens, HIMAP4 , B factor, C5, AAT and H factor, which had a lower level of mRNA expression in HCC relative to the adjacent noncancerous tissues. AAT was found to inhibit angiogenesis and tumour growth, its downregulation in cancer may associate with tumour progression (Huang *et al*, 2004a). Four other antigens associate with the inhibition of immune responses, and hence may facilitate tumour escaping.

Nine of the antigens associated with an HCC-related serological response, CAGE, SR140 protein, HSPCA, PSMD1, HDAC2, RNPC2, galectin 4, transgelin 2 and PSMA7, were overexpressed in HCC. Transgelin 2, which has been reported to be overexpressed in gastric cancer (Ryu *et al*, 2003), was found to be upregulated in 11 of 16 HCC patients. This protein is thought to be involved in cell proliferation and migration, suggesting that its overexpression may be implicated in tumour progression. The fact that transgelin 2 was overexpressed and detected at a high rate (69%) in HCC specimens has made this molecule a candidate HCC marker for diagnosis. HDAC2, which is considered as a key element in the dynamic regulation of many genes regulating cellular proliferation and differentiation during carcinogenesis (Cress and Seto, 2000), was found to be upregulated in seven of 16 HCC patients, suggesting that its overexpression may have aetiologic significance. A recent report showed that elevated HDAC2 mRNA expression was observed in 82% of 57 human colonic cancer patients (Zhu *et al*, 2004). In addition, a large number of studies have shown that HDAC inhibitors can effectively arrest and revert transformation of some cells and block the formation of tumours in rodent models. These observations suggest that HDAC inhibitors may be candidate drugs in therapy for human cancer (Cress and Seto, 2000). PSMA7, which was found to be overexpressed in seven of 16 HCC patients, is a subunit of proteasome, and has been shown to interact specifically with the hepatitis B virusxprotein, a protein critical to viral replication (Zhang *et al*, 2000). It is also involved in regulating hepatitis virus C internal ribosome entry site activity, which is essential for viral replication (Kruger *et al*, 2001). In consideration of the close correlation of hepatitis with HCC, upregulation of PSMA7 in HCC may play an important role in aetiopathogenesis of HCC. PSMD1, which is involved in processing of class 1 MHC peptide (Coux *et al*, 1996), was found to be overexpressed in six of 16 HCC patients. However, its association with tumour is less obvious. Transcripts encoding 2 other antigens with cancer-related serological profiles, HSPCA and galectin 4, have been reported to be overexpressed in 45% (20 of 45) (Huang *et al*, 2004b) and 80% (four of five) (Kondoh *et al*, 1999) in HCC patients, respectively; their upregulation in HCC may associate with occurrence and progression of tumour. As regulator for pre-mRNA splicing, overexpression of RNPC2 and SR140 protein may also be of aetiological significance in HCC.

In the attempt to find HCC tumour markers among our SEREX-identified 30 antigens, the mRNA expression level was quantitatively measured by cDNA microarray and real-time PCR, and there were nine genes upregulated, five genes downregulated and 16 genes unaltered. These results indicate that the immunogenicity of autoantigens in cancer patients is not closely correlated with their mRNA expression levels. An immune response to apparently unaltered gene products in cancer patients might be due to the crossreaction of the antibodies generated against the mutated product in cancer patients with the unaltered wild-type gene product (Scanlan *et al*, 1998). Alternatively, the bystanding help effect in cancer patients may activate autoantigens to induce antibody response.

Overall, our SEREX-identified new tumour antigens in HCC add more information in HCC immunome, which may help in understanding the HCC tumorigenesis. And the SEREX-defined CT antigen CAGE in HCC may be a potential candidate for tumour vaccine design and transgelin 2 might be an HCC tumour marker for diagnosis.

## Figures and Tables

**Table 1:** Genes cloned from hepatocellular carcinoma by SEREX

**No.**	**GenBank access no.**	**Gene**	**Clone repeat**	**Remark**
1	NM_000186	H factor 1	4	A component of Complement System
2	NM_001710	B factor	1	A component of Complement System
3	NM_000295	AAT	1	Alpha 1-antitrypsin
4	NM_001735	C5	1	Complement component 5
5	NM_013450	BAZ2B	1	Involved in chromatin remodelling
6	NM_001527	HDAC2	1	Regulating chromatin structure
7	XM_031553	SR140 protein	5	Involved in the regulation of alternative splicing
8	NM_004719	SFRS2IP	1	Splicing pre-mRNA
9	NM_004902	RNPC2	1	Splicing factor
10	NM_016224	Sorting nexin 9	1	Involved in intracellular trafficking
11	NM_001012	RPS8	5	Ribosomal protein S8
12	NM_007065	CDC37	1	Molecular chaperone
13	NM_005348	HSPCA	1	Molecular chaperone
14	NM_002807	PSMD1	2	A non-ATPase subunit
15	NM_005176	ATP5G2	1	Subunit c of ATP synthase
16	NM_002792	PSMA7	1	Proteasome subunit, alpha-type,7
17	NM_005842	SPRY2	1	Regulating epidermal growth factor receptor/mitogen-activated protein kinase
18	NM_004987	PINCH	2	Likely involved in integrin signalling
19	NM_002415	MIF	1	Migration inhibitory factor
20	NM_014961	RIPX	4	Rap2 interacting protein ×
21	NM_006499	Galectin 8	1	Involved in modulating cell–cell and cell–matrix interactions
22	NM_006149	Galectin 4	2	Involved in modulating cell–cell and cell–matrix interactions
23	NM_006346	PIBF1	1	Progesterone-induced blocking factor 1
24	NM_003564	Transgelin 2	2	Involving in cell proliferation and migration
25	XM_042860	KIAA0379	1	Hypothetical protein
26	AF543495	Melanoma-associated antigen	1	Function unknown
27	NM_016436	HCA58	2	Function unknown
28	NM_032639	FAPP2	3	Phosphoinositol 4-phosphate adaptor protein-2
29	NM_018326	HIMAP4	1	*Homo sapien* immunity-associated protein 4
30	AY039237	CAGE	1	CT antigen
